# ERAP1 deficient mice have reduced Type 1 regulatory T cells and develop skeletal and intestinal features of Ankylosing Spondylitis

**DOI:** 10.1038/s41598-018-30159-5

**Published:** 2018-08-20

**Authors:** Yuliya Pepelyayeva, David P. W. Rastall, Yasser A. Aldhamen, Patrick O’Connell, Sandra Raehtz, Fadel S. Alyaqoub, Maja K. Blake, Ashley M. Raedy, Ariana M. Angarita, Abdulraouf M. Abbas, Cristiane N. Pereira-Hicks, Sarah G. Roosa, Laura McCabe, Andrea Amalfitano

**Affiliations:** 1 Department of Microbiology and Molecular Genetics, Michigan State University, East Lansing, MI 48824 USA; 2 Department of Pediatrics, College of Osteopathic Medicine, Michigan State University, East Lansing, MI 48824 USA; 3 Department of Physiology, Michigan State University, East Lansing, MI 48824 USA; 4 Department of Radiology, Michigan State University, East Lansing, MI 48824 USA; 5 Biomedical Imaging Research Center, Michigan State University, East Lansing, MI 48824 USA

## Abstract

Ankylosing spondylitis (AS) is a prototypical sero-negative autoimmune disease that affects millions worldwide. Single nucleotide polymorphisms in the Endoplasmic Reticulum Aminopeptidase 1 (ERAP1) gene have been linked to AS via GWAS studies, however, the exact mechanism as to how ERAP1 contributes to pathogenesis of AS is not understood. We undertook µCT imaging and histologic analysis to evaluate bone morphology of the axial skeletons of ERAP1^−/−^ mice and discovered the hallmark skeletal features of AS in these mice, including spinal ankylosis, osteoporosis, and spinal inflammation. We also confirmed the presence of spontaneous intestinal dysbiosis and increased susceptibility to Dextran Sodium Sulfate (DSS)-induced colitis in ERAP1^−/−^ mice, however the transfer of healthy microbiota from wild type mice via cross-fostering experiments did not resolve the skeletal phenotypes of ERAP1^−/−^ mice. Immunological analysis demonstrated that while ERAP1^−/−^ mice had normal numbers of peripheral Foxp3^+^ Tregs, they had reduced numbers of both “Tr1-like” regulatory T cells and tolerogenic dendritic cells, which are important for Tr1 cell differentiation. Together, our data suggests that ERAP1^−/−^ mice may serve as a useful animal model for studying pathogenesis of intestinal, skeletal, and immunological manifestations of Ankylosing Spondylitis.

## Introduction

Ankylosing Spondylitis (AS) is a highly heritable autoimmune disease with an estimated prevalence of 1% world-wide^1^. AS is characterized by early-onset fusion of bones in the spine and pelvis, referred to as ankylosis^2^. Many AS patients also develop osteopenia or osteoporosis in both the axial and the peripheral skeletons, predisposing these individuals to pathologic fractures^3,4^. Over 40 years ago, the genetic association of the Human Leukocyte Antigen - HLA-B*27 allele with AS was found, directly implicating immune mechanisms in AS susceptibility^5^. Despite this knowledge the exact mechanism underlying pathogenesis of inflammation and spinal fusions in AS is still not understood.

For example, while over 90% of AS patients have an HLA-B*27 variant, only 1–5% of individuals carrying HLA-B*27 develop AS, suggesting that additional risk factors must be present^2^. While HLA-B*27 is thought to contribute 23% of the genetic risk for AS, there are strong epistatic gene-gene interactions between the presence of specific *ERAP1* variants and HLA-B*27^6^. As one of ERAP1’s primary functions is to trim peptides prior to their loading onto MHC-I molecules, epistasis between ERAP1 and HLA alleles supports the hypothesis that deviations in antigen presentation pathways may underlie AS pathogenesis^7,8^. Additionally, our work, and that of others has also implicated ERAP1 in the suppression of innate and adaptive immune responses^9–11^. Adding to this genetic and immunologic complexity, gastrointestinal abnormalities are also thought to be involved in AS pathogenesis^12^. Patients with colitis are three times more likely to develop clinical AS,^13^ and up to 60% of AS patients manifest microscopic inflammatory damage to their gut mucosa^4,12^. It has also been found that AS patients have altered microbiota in the terminal ilium^14^, indirectly suggesting that microbial interactions with the intestinal immune system may contribute to extra-intestinal immune abnormalities and pathogenesis of AS^15–17^.

We wished to investigate the potential role of ERAP1 in the skeletal and intestinal pathogenesis of AS by critically evaluating the skeletal, intestinal and immunological phenotypes of ERAP1^−/−^ mice. Here we show that deficiency of ERAP1 was sufficient to cause a spontaneous and rapid onset of spinal ankylosis, calcification of the anterior longitudinal ligament (ALL), sacro-iliac (SI) erosions, and systemic osteoporosis in mice, making this a valuable model to use for studying the pathogenesis of these skeletal abnormalities that are also found in human AS. Moreover, ERAP1^−/−^ mice developed intestinal features including spontaneous dysbiosis and increased susceptibility to dextran sodium sulfate (DSS)-induced colitis, paralleling intestinal features of AS. In our survey of the immune system of ERAP1^−/−^ mice, we evaluated two major populations of suppressive T cells including Foxp3^+^Tregs and Type 1 regulatory T cells (Tr1s)^18^. While splenic Foxp3^+^Treg numbers and function did not differ between WT and ERAP1^−/−^ mice, we detected a significant reduction in the numbers of CD3^+^CD4^+^Foxp3^−^IL10^+^ “Tr1-like” cells present peripherally in ERAP1^−/−^ mice. In addition, we observed reduced numbers of tolerogenic dendritic cells (tDCs), which are thought to be important for Tr1 differentiation and function^19^.

## Results

### ERAP1 deficiency causes spinal ankylosis and osteoporosis

**Figure 1:**
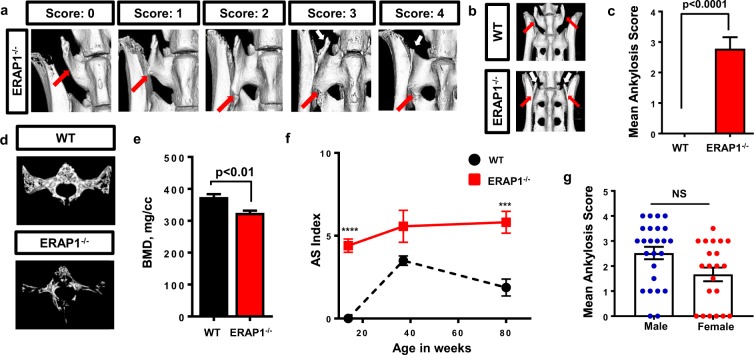
µCT analysis of the axial skeleton. The spines from ERAP1^−/−^ and WT mice were harvested and analyzed with µCT. (**a**) Scoring system with representative images from ERAP1^−/−^ mice. (**b**) Representative isosurface images, demonstrating spinal ankylosis of the L6 to S1 vertebra in ERAP1^−/−^ mice. Red arrows indicate the L6 vertebra. White arrows indicate remnants of the transverse process of L6. 14-weeks-old WT (n = 10) and ERAP1^−/−^ (n = 16) male and female mice were assessed for ankylosis bilaterally. Scores were averaged, and Mean Ankylosis Scores were plotted on bar graph (**c**). (**d**) Representative isosurface images of the trabecular bone of S1 vertebra. (**e**) Graph depicting bone mineral density (BMD) of the trabecular bone of S1 vertebra of 14-week-old female mice (n = 5). (**f**) AS index was calculated by combining the fusion and an assigned BMD score, as described in methods, and graphed as a function of time. (**g**) Fusion scores of 14-weeks-old ERAP1^−/−^ mice graphed as function of sex. Values on graphs are the mean ± SEM. p values calculated by student’s t-test, where ***p < 0.001, ****p < 0.0001 and NS- not significant.

*ERAP1* SNPs have been genetically linked to increased susceptibility of AS^8^. We first surveyed the spinal morphology of ERAP1^−/−^ mice for skeletal manifestations observed in human patients, such as sacroiliitis, syndesmophyte bridging, joint erosions and/or osteoporosis of the spine^4^. We performed μCT analysis of the axial skeletons of 14-weeks-old WT and ERAP1^−/−^ mice and observed that ERAP1^−/−^ mice exclusively developed spinal ankylosis between the transverse processes of L6 (lumbar vertebrae 6) and S1 (sacral vertebrae 1) [Fig. 1a–c]. We developed a scoring system ranging from 0 to 4, with 0 representing a normal L6-S1 joint, and 4 representing a joint architecture in which an abnormal syndesmophyte was extended along the full border of the iliac bone and was completely fused with S1 [Fig. 1a]. The left and right sides of this joint were scored independently, and the scores were averaged to get the mean ankylosis score for each individual mouse. Our detailed skeletal survey of ERAP1^−/−^ mice showed that ERAP1^−/−^ mice not only exclusively developed spinal ankylosis between L6 and S1 [Fig. 1b,c], but also developed other hallmarks of human AS, such as iliac erosions [Supplemental Fig. 1a,b] and calcification of the anterior longitudinal ligament [Supplemental Fig. 1c]. While there was no significant difference in the severity of fusions between male and female mice, we observed a clear trend in reduced severity of fusions and reduced prevalence of fusions in female mice, a result that parallels known gender differences for the human disease^1^ [Fig. 1g].

To assess ERAP1^−/−^ mice for osteoporosis of the spine, we analyzed the trabecular bone morphology of 14-week-old ERAP1^−/−^ mice utilizing quantitative micromorphometry and MicroView software. The bone mineral density (BMD) of the S1 vertebral body in ERAP1^−/−^ mice was significantly reduced compared to WT [Fig. 1d,e]. Other measures of trabecular bone structure including bone volume fraction (BV/TV %), bone mineral content (BMC), trabecular number (Tb.N.), trabecular thickness (Tb.Th.), and trabecular spacing (Tb.Sp.) confirmed the presence of an osteoporotic phenotype in ERAP1^−/−^ mice [Supplemental Fig. 1e–i]. We observed similar findings in the trabecular bone of L6 vertebral body and femurs of ERAP1^−/−^ mice [data not shown]. Assessment of the trabecular bone of S1 vertebrae at different ages revealed that ERAP1^−/−^ mice failed to gain maximal bone volume fraction at 14 weeks of age, which continued to be significantly lower compared to age-matched WT mice throughout their lifetime [Supplemental Fig. 1d]. We observed the same trends in other trabecular measures [data not shown]. We developed a quantitative AS severity scoring system (AS Index), which combined ankylosis score and an assigned bone mineral density score, where 325–300 mg/cc received score 1, 299–275 – 2, 274–250 – 3 and <250 – 4. The AS index was also used to evaluate disease progression between ERAP1^−/−^ and WT mice over time [Fig. 1f]. The AS index was significantly increased in ERAP1^−/−^ mice as compared to WT at 14 and 80 weeks of age, with some evidence of progression in severity of this score over time. This is the first report, to our knowledge, identifying a robust animal model in which young animals simultaneously present with spontaneous axial ankylosis and reduced bone density.

### Deficiency of ERAP1 results in recruitment of immune cells to spinal joints

**Figure 2:**
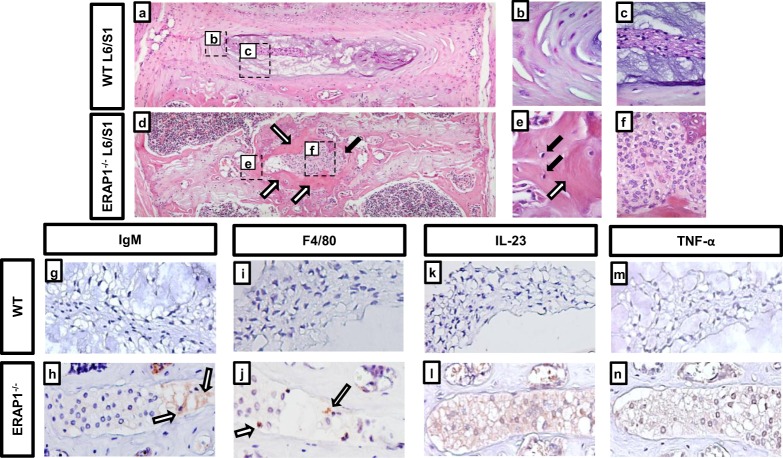
Histopathologic evaluation of ankylosed intervertebral joints. Spines from 84-week-old WT and ERAP1^−/−^ female mice (n = 3) were harvested, fixed, and stained. (**a**) Representative 10x H&E image of a WT L6/S1 joint demonstrating healthy annulus fibrosus (**b**), and nucleus pulposus (**c**). (**d**) Representative H&E of an ERAP1^−/−^ L6/S1 joint demonstrating disruption of the disc by a large infiltrative mass of cells (black arrow and f) and ectopic bone (white arrows and e) within the joint. (**e**) 60x magnification of intervertebral bone formation with black arrows demonstrating osteocytes. Spines from 14-week-old male and female mice (n = 3) were harvested, fixed, and stained with anti-IgM, IL-23, TNFα, and F4/80 antibodies. Immunohistochemistry representative images at 20X magnification demonstrating IgM + deposits (white arrows) within the nucleus pulposus of L6/S1 joint in ERAP1^−/−^ (**h**), but not in WT mice (**g**). Some cellular infiltrates in the nucleus pulposus within the L6/S1 joints of ERAP1^−/−^ mice stained positive for F4/80 + (white arrows), indicating macrophages (**j**), but not in WT mice (**i**). IL-23 positive staining of the L6/S1 nucleus pulposus infiltrate in ERAP1^−/−^ (**l**), but not in WT **(k)**. TNFα positive staining of the nucleus pulposus in ERAP1^−/−^ (**n**) but not in WT L6/S1 joint (**m**).

We wished to evaluate the structure and integrity of the ankylosed joints in ERAP1^−/−^ mice first using H&E staining. L6/S1 joints of WT mice demonstrated a normal architecture of concentrically lamellated annulus fibrosus (AF) and nucleus pulposus (NP), with characteristic cells and matrix [Fig. 2a–c]. In contrast, the ankylosed L6/S1 joints from age-matched ERAP1^−/−^ mice demonstrated severe pathology [Fig. 2d–f]. The NP structure was replaced by a dense cellular infiltrate dominated by mononuclear cells with large cytoplasm and heterochromatic nuclei [Fig. 2f]. The structure of AF was disrupted, and NP/AF interface was surrounded by ectopic bone [white arrows in Fig. 2e] and resident osteocytes [black arrows in Fig. 2e]. To determine if the infiltrative mononuclear cells were of immune origin, we performed immunohistochemistry for IgM, F4/80, CD3, IL23, and TNFα markers. IgM, F4/80, IL23 and TNFα positive staining was detected in the NP of ERAP1^−/−^ mice by 14 weeks, compared to age-matched WT mice [Fig. 2g–n]. IgM + and F4/80 + staining suggests presence of IgM deposits [Fig. 2h] and macrophages [Fig. 2j] in the NP, respectively. CD3 staining was negative, suggesting absence of T cells (data not shown). The cytoplasm of large mononuclear cells in the NP of ERAP1^−/−^ mice stained positive for IL-23 [Fig. 2l] and TNFα [Fig. 2n] signifying the presence of abnormal local inflammatory responses in the L6/S1 intervertebral disc (IVD) of ERAP1^−/−^ mice.

### ERAP1 deficient mice have increased susceptibility to chemically-induced colitis

**Figure 3:**
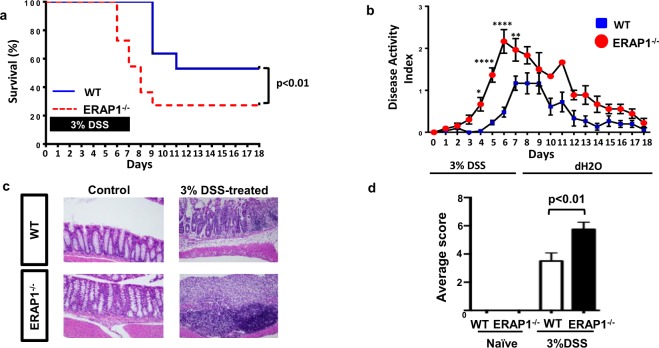
Induction of chemically-induced colitis. 8-week-old male WT and ERAP1^−/−^ mice (n = 11) were treated with a 3% DSS solution in drinking water for 7 days, followed by regular drinking water for 11 days. The DSS solutions were made fresh at day 0 and 3 and left until day 7. Mice were monitored until day 18 after the start of DSS. (**a**) Survival plot of WT and ERAP1^−/−^ DSS-treated mice. Differences in group survival were analyzed with the Kaplan-Meier test. Disease Activity Index of WT and ERAP1^−/−^ mice was determined as described in materials and methods and graphed in (**b**). Two-way ANOVA with Bonferroni test were used to determine significant differences. (**c**) Representative images of H&E staining for histopathological changes in colon tissue of naïve and DSS-treated WT and ERAP1^−/−^ mice (n = 4). Semiquantitative scoring of histopathology of colons was performed, as described in Materials and Methods section and plotted as bar graph in (**d**). Data are expressed as the mean ± SEM. p values calculated by student’s t-test, where *p < 0.05, **p < 0.01 and ****p < 0.0001. Data are representative of three independent experiments, with similar results.

In addition to skeletal involvement, AS patients are known to have an increased susceptibility to inflammatory bowel disease (IBD), with 5–10% of AS patients developing IBD^4^, and up to 60% of AS patients having evidence of subclinical gut inflammation in the terminal ileum^4,12^. To determine if ERAP1 functions impact the intestinal system to a degree that may influence the development and severity of colitis, we assessed the mortality rate of age- and sex-matched WT and ERAP1^−/−^ mice after 3% DSS challenge, which is known to induce intestinal inflammation and colitis by altering the integrity of the intestinal epithelial barrier^20,21^. We observed a significantly increased mortality rate of DSS-treated ERAP1^−/−^ mice, beginning as early as 6 days after DSS challenge as compared to identically treated WT mice [Fig. 3a]. Only 27% of ERAP1^−/−^ mice survived the challenge as compared to 53% survival in the WT controls (p < 0.01) [Fig. 3a]. Mice were also monitored for weight loss, stool consistency, and rectal bleeding scores, all of which were used to calculate an overall colitis disease activity index (DAI). The DAI was significantly increased in DSS-treated ERAP1^−/−^ mice, as compared to WT mice starting on day 4 following DSS administration (p < 0.05) [Fig. 3b]. Histologic colon sections taken from WT and ERAP1^−/−^ naïve mice on day 7 showed limited evidence of lymphocytic infiltration of the mucosa and submucosa, and little to no evidence of focal erosions or ulcerations [Fig. 3c]. In contrast, colonic sections of DSS-treated ERAP1^−/−^ mice displayed extensive ulceration and inflammation with foci of proliferating lymphocytes [Fig. 3c]. Utilizing a previously described semi-quantitative scoring system^22^, we confirmed the presence of significantly (p < 0.01) increased inflammatory signs of colitis in DSS-treated ERAP1^−/−^ mice, as compared to WT controls [Fig. 3d]. These results suggest that the changes observed in DSS-challenged ERAP1^−/−^ mice were not due to anatomic differences prior to the challenge, but rather due to abnormal immune responses to colonic irritation, findings consistent with other models of IBD^20,21^.

### ERAP1 deficient mice develop spontaneous gut dysbiosis

**Figure 4:**
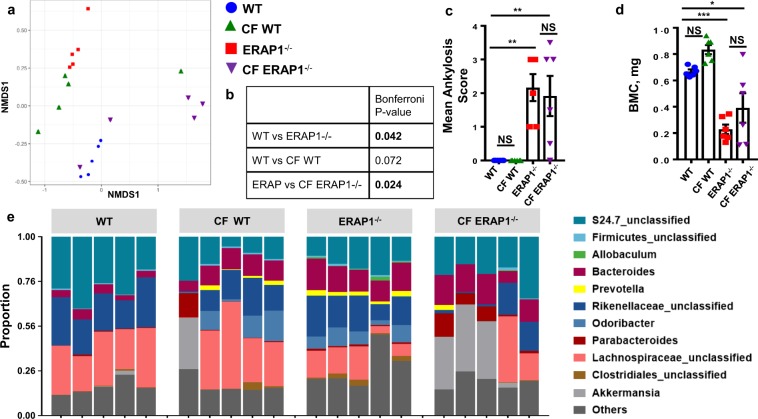
Evaluation of gut microbial composition and cross-foster experiments. 16S rRNA phenotyping was performed with illumina on fecal samples of 14-week-old cross-fostered male and female mice (n = 5). (**a**) A principal component analysis of the composition of the gut microbiome was represented by summarizing OTU abundances into Bray-Curtis dissimilarities and performing a non-metric multidimensional scaling (NMDS) ordination. To quantify the differences between the groups, PERMANOVA was performed. (**b**) Table summarizing Bonferroni p-values. Spines of 14-week-old cross-fostered male and female mice (n = 6) were surveyed for spinal fusions and osteoporosis using μCT. Mean Ankylosis Score (**c**) and BMC (**d**) of the trabecular bone of S1 vertebra graphed as the mean ± SEM. Statistical significance was determined using One Way ANOVA, and Tukey post hoc test, where *p < 0.05, **p < 0.01, ***p < 0.001, and NS – not significant. Data are representative of three independent experiments, with similar results. (**e**) Relative abundance of genera in fecal samples of WT, CF WT, ERAP1^−/−^, and CF ERAP1^−/−^ mice. Genus-level OTUs significantly different between WT and ERAP1^−/−^ fecal samples are summarized in Supplemental Table 1. The microbiome data is a representative of two independent experiments with similar results.

While there is strong evidence suggesting that the intestinal microbiota plays a role in modulating immune diseases that affect the intestine, such as IBD^23^, there is less information available in regard to the direct role of the gut microbiota in causing arthritic autoimmune diseases, such as AS^15,24^. Recently, dysbiosis of the terminal ileum has been noted in AS patients, suggesting that the microbiota may play a role in AS^14^. We undertook genetic phenotyping of the fecal microbiomes of similarly-housed ERAP1^−/−^ and WT mice to determine if spontaneous dysbiosis was present in ERAP1^−/−^ mice. Microbial communities present in the fecal samples of mice were determined by analysis of the V4 hypervariable region of the 16 S rRNA gene. A non-metric multidimensional scaling analysis demonstrated that ERAP1^−/−^ mice have a significantly altered fecal microbiome composition (p < 0.05), as compared to similarly-housed WT mice [Fig. 4a,b]. Plotting of aggregated operational taxonomic units (OTUs) into phylum-level taxa revealed that fecal samples derived from 14-week-old ERAP1^−/−^ mice were significantly enriched for *Cyanobacteria* (p < 0.05) and *Actinobacteria* (p < 0.05) [Supplemental Fig. 2]. Genus-level analysis of ERAP1^−/−^ samples revealed enrichment of *Prevotella*, *Odoribacter*, *Bacteroides*, and to lesser extent *YS2, Parabacteroides* and *Clostridiales* as compared to fecal samples from similarly-housed and age-matched WT mice [Fig. 4e and Supplemental Table 1]. In addition, ERAP1^−/−^ mice were deficient in the bacterial genus *Lachnospiraceae* and to lesser extent *Christensenellaceae* and *S24.7* [Fig. 4e and Supplemental Table 1]. These results confirm that ERAP1^−/−^ mice exhibit another phenotype found in AS patients, namely spontaneous intestinal dysbiosis^14^.

We next wished to determine if the intestinal microbiome plays a role in the development of skeletal pathology in ERAP1^−/−^ mice. We undertook cross-fostering experiments, which have been shown to be an effective method of inducing an early and maintained shift in the commensal microbiota to that of a nursing mother^25^. Fecal 16S rRNA phenotyping of ERAP1^−/−^ mice cross-fostered to WT dams (CF ERAP1^−/−^) showed significant changes in their microbiome compared to ERAP1^−/−^ samples (p < 0.05) [Fig. 4b], with normalization of *Cyanobacteria* and *Actinobacteria* phyla [Supplemental Fig. 2]. We also observed correction of *Prevotella*, *Odoribacter*, *Clostridiales*, *Lachnospiraceae, YS2* and *S24.7* genus, but not *Bacteroides, Parabacteroides*, and *Christensenellacea* [Fig. 4e and Supplemental Table 1]. Despite these significant alterations in the intestinal microbiota, μCT analysis of CF ERAP1^−/−^ did not show any changes in their ultimate development of ankylosis [Fig. 4c] or osteoporosis [Fig. 4d] during the time frame of these experiments. Similarly, CF ERAP1^−/−^ mice still had TNFα, IL-23, and F4/80 positive staining, but not IgM in the NP of their L6/S1 disc spaces [Supplemental Fig. 3]. Confirming the utility of cross-fostering, WT pups cross-fostered to ERAP1^−/−^ dams (CF WT), indeed clustered similarly to ERAP1^−/−^ samples [Fig. 4a]. Four cross-fostered mice created a cluster of their own, with biggest contribution from *Akkermansia* genus, increasing the variability within cross-foster groups. Despite transfer of the aberrant microbiota derived from ERAP1^−/−^ mice to CF WT mice, no ankylosis or significant changes in the bone density measures were observed in the latter [Fig. 4c,d].

### ERAP1 deficient mice have reduced numbers of suppressor “Tr1-like” cells

**Figure 5:**
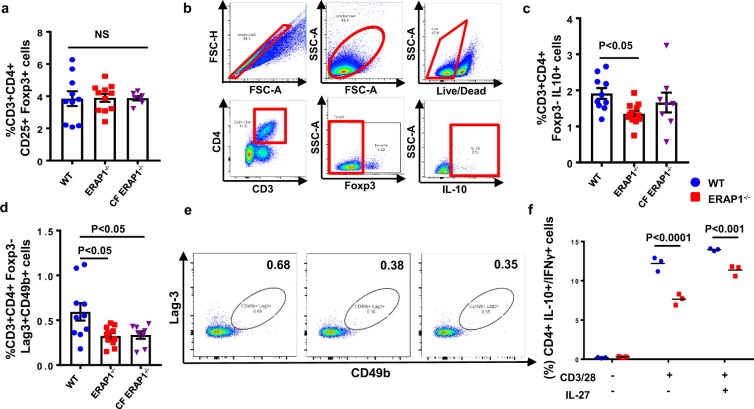
Evaluation of splenic Tr1 cells. Spleens from 14-week-old male and female WT (n = 10), ERAP1^−/−^ (n = 12) and CF ERAP1^−/−^ (n = 8) mice were harvested, processed, stained and analyzed by flow cytometry. (**a**) Frequency of CD3^+^CD4^+^CD25^+^Foxp3^+^ Treg cells in WT, ERAP1^−/−^, and CF ERAP1^−/−^ mice graphed. (**b**) Gating strategy for Tr1 cells is highlighted in red. (**c**) Frequency of IL-10 producing CD3^+^CD4^+^Foxp3^−^ “Tr1-like” cells in WT, ERAP1^−/−^, and CF ERAP1^−/−^ mice graphed. (**d**) Graph depicting frequencies of CD3^+^CD4^+^Foxp3^−^Lag-3^+^CD49b^+^ cells in WT, ERAP1^−/−^, and CF ERAP1^−/−^ mice, with representative images in (**e**). Statistical significance was determined using One Way ANOVA, and Tukey post hoc test. Naïve CD4^+^ T cells were isolated and cultured for 4 days in the presence or absence of CD3/28 and IL-27 to induce Tr1 cell differentiation as described in methods. Flow cytometry analysis measuring relative abundance of IL-10 and IFNγ producing CD4^+^ T cells is graphed on scatter plot (**f**). Two-way ANOVA with Tukey post hoc test was used for determination of statistical significance. Data are representative of two independent experiments, with similar results. NS - not significant.

We have previously demonstrated that ERAP1^−/−^ mice exhibit exaggerated innate and adaptive immune responses to antigenic stimuli, suggesting that they might lack a global immune suppressive function^10^. Two major populations of suppressive T cells include Foxp3^+^ Tregs and Tr1 cells^18^. To investigate if ERAP1 function impacts Treg number or function, we first evaluated the number of conventional Foxp3^+^CD4^+^CD25^+^ Tregs in the spleens of ERAP1^−/−^ and WT mice but identified no significant differences between them [Fig. 5a]. We also evaluated the production of IL-10 and TGF-β, as well as analyzed the immune suppressive functional activity of conventional Tregs in cell culture systems, and again observed no differences in these activities between WT and ERAP1^−/−^ derived Tregs [data not shown]. Additionally, we surveyed WT and ERAP1^−/−^ spleens for numbers of Th17 cells, which have been implicated in playing a role in AS^26^, but observed no differences [data not shown].

Although less-studied than conventional Tregs, Tr1 cell functions have been implicated in multiple autoimmune diseases, including insulin-dependent diabetes mellitus (IDDM)^27^, Multiple Sclerosis (MS)^28^, and IBD^29^, all of which have associations with ERAP1 polymorphisms^30^. In contrasts to our analysis of Tregs, spleens derived from ERAP1^−/−^ mice contained significantly reduced numbers of CD3^+^CD4^+^Foxp3^−^IL10^+^ “Tr1-like” cells, as compared to splenocyte populations derived from WT mice (p < 0.05) [Fig. 5b,c]. These results were also confirmed when using two cell surface markers, LAG3 and CD49b, considered to be important in identifying human and murine Tr1 cells by flow cytometry analysis^18^ [Fig. 5d,e].

We wished to assess whether reduced levels of peripheral “Tr1-like” cells in ERAP1^−/−^ mice were due to reduced ability of naïve T cells to differentiate into Tr1 cells. Naïve CD4^+^ T cells derived from WT and ERAP1^−/−^ mice were isolated and cultured in the presence of anti-CD3, anti-CD28 antibodies, and recombinant IL-27 to induce Tr1 differentiation^31^. After 4 days of anti-CD3, anti-CD28 antibodies and recombinant IL-27 stimulation, there were significantly (p < 0.001) reduced numbers of “Tr1-like” cells (CD4^+^IL-10^+^/IFN-γ^+^ cells) derived from naïve CD4^+^ T cells of ERAP1^−/−^ mice, as compared to identical cultures derived from WT mice [Fig. 5f]. Finally, CF ERAP1^−/−^ mice also had significantly (p < 0.05) reduced number of splenic “Tr1-like” cells as compared to WT mice [Fig. 5d], indicating that transfer of microbiota from WT mice also did not correct the reduced Tr1 numbers noted in ERAP1^−/−^ mice.

### ERAP1^−/−^ mice have reduced numbers of tolerogenic dendritic cells

**Figure 6:**
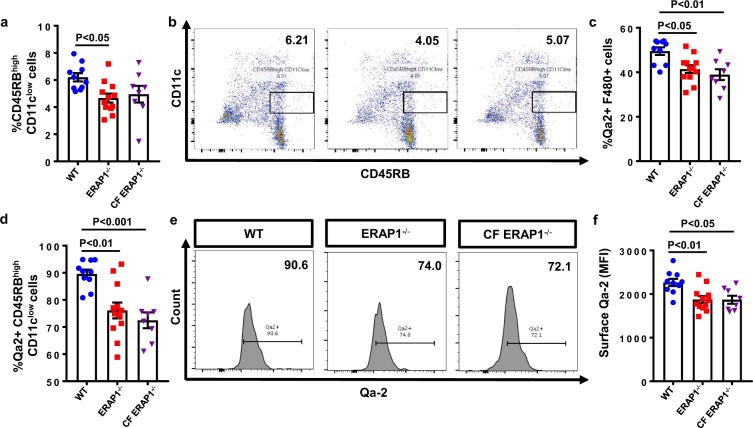
Evaluation of tolerogenic dendritic cells (tDCs). Spleens from 14-week-old male and female WT (n = 10), ERAP1^−/−^ (n = 12), and CF ERAP1^−/−^ (n = 8) mice were harvested, processed, stained and analyzed by flow cytometry. (**a**) Relative frequencies of tolerogenic CD45RB^high^ CD11c^low^ dendritic cells (tDCs) in WT, ERAP1^−/−^, and CF ERAP1^−/−^ spleens plotted on scatter plot bar graph, with representative images in (**b**). (**c**) Graph of relative frequencies of Qa-2 expressing F4/80 + macrophages. (**d**) Graph, depicting percent of Qa-2 expressing CD45RB^high^ CD11c^low^ tDCs in WT, ERAP1^−/−^, and CF ERAP1^−/−^ mice. (**e**) Representative histogram images demonstrating relative abundance of Qa-2 positive tDCs. (**f**) Graph depicting Mean Fluorescence Intensity (MFI) of surface Qa-2 on tDCs from WT, ERAP1^−/−^, and CF ERAP1^−/−^ mice. Statistical significance was determined using One Way ANOVA, and Tukey post hoc test. Data are representative of two independent experiments, with similar results.

CD45RB^high^ CD11c^low^ tDCs are thought to be required for optimal generation of Tr1 cells from naïve CD4^+^ T cells^19^, so we wished to assess the number and phenotype of these cells in ERAP1^−/−^ mice. Splenocytes derived from ERAP1^−/−^ had significantly reduced (p < 0.05) numbers of CD45RB^high^ CD11c^low^ tDCs as compared to splenocytes derived from WT mice [Fig. 6a,b]. The non-classical HLA-G molecule is thought to be important for differentiation of T cells into Tr1 cells in humans via its interactions between tDCs and naïve T cells^32,33^. It is well known that ERAP1 functions directly influence classical MHC-I surface levels^34–37^. In addition, it has been reported that siRNA mediated loss of ERAP1 can prevent HLA-G upregulation in trophoblast cell cultures^38^. We therefore measured surface levels of Qa-2 (the murine homolog of HLA-G) on antigen-presenting cells (APCs) of ERAP1^−/−^ mice. Intriguingly, we observed reduced numbers of Qa-2+ macrophages (p < 0.05) [Fig. 6c] and tDCs (p < 0.01) in the spleens of ERAP1^−/−^ mice [Fig. 6d,e]. Mean fluorescence intensity (MFI) of Qa-2 was also significantly reduced in the splenic macrophages [data not shown] and tDCs (p < 0.01) of ERAP1^−/−^ mice [Fig. 6f]. Percent of Qa-2 positive tDCs and macrophages, as well as the MFI surface levels of Qa-2 on tDCs also remained significantly reduced in CF ERAP1^−/−^ mice [Fig. 6c,d,f]. Aberrant number of tDCs and Qa-2 surface levels suggest a possible mechanism responsible for the reduced numbers of Tr1 cells observed in ERAP1^−/−^ mice, as further elucidated in the Discussion.

## Discussion

Here we show that global deficiency of the *ERAP1* gene caused development of spontaneous axial ankylosis, spinal inflammation, and progressive systemic osteoporosis in mice. Furthermore, similar to AS patients, who have increased susceptibility to IBD^4^ and dysbiosis in the terminal ileum^14^, ERAP1^−/−^ mice also developed intestinal phenotypes manifested as increased susceptibility to chemically induced colitis and spontaneous gut dysbiosis^14^. Together, these findings suggest that ERAP1^−/−^ mice are an important and useful new animal model for studying the pathogenesis of the most important skeletal^2–4,39^ and intestinal manifestations found in AS patients^12,14^. ERAP1^−/−^ mice can also be a useful tool for testing therapeutic agents targeting skeletal, immune, and intestinal manifestations of AS. In addition, ERAP1^−/−^ mice are a unique animal model which can be used as tool for studying interactions of the gut-bone-immune axis.

We and others have identified that ERAP1^−/−^ mice manifest exaggerated innate and adaptive immune responses to a number of stimuli as compared to WT mice, suggesting loss of global immune suppressive functions^9–11,34–36^. While previous reports indirectly noted that Foxp3^+^ Tregs are perturbed in AS^40,41^, we did not identify differences in the number nor the function of Foxp3^+^ Tregs in the periphery of ERAP1^−/−^ mice. While we cannot rule out entirely that a function of Foxp3^+^ Tregs may be influenced by loss of ERAP1, our analysis readily confirmed that another major class of regulatory T cells, the “Tr1-like” cells, were significantly reduced in ERAP1^−/−^ mice. Tr1 cells are characterized by their ability to secrete high levels of IL-10 and TGF-β in the absence of Foxp3 expression, and their ability to suppress T cell and APC responses in the periphery^18^. We further identified that the number of tDCs was also significantly reduced in the spleens of ERAP1^−/−^ mice. It is known that tDCs induce naïve CD4^+^ T cells to differentiate into Tr1 cells^19^. Namely, HLA-G expression on tDCs and its interaction with Ig-like transcript 2 (ILT-2) and ILT-4 receptors on naïve CD4^+^ T cells are thought to be important for tDC maturation and differentiation of Tr1 cells^32,33^. While ERAP1’s role in classical MHC-I surface expression is well known, shRNA mediated loss of ERAP1 in human trophoblasts was also shown to reduce non-classical MHC-I HLA-G surface levels^38^. Our analysis showed that Qa-2, the murine homolog of HLA-G, was also significantly reduced on tDCs from ERAP1^−/−^ mice. Together our results suggest that the possible mechanism responsible for altered Tr1 differentiation in ERAP1^−/−^ mice may be due to reduced tDC numbers and surface levels of Qa-2. Further studies are needed to better understand the mechanism via which ERAP1 mediates Tr1 cell differentiation, and what role these cells may play in the pathogenesis of the phenotypes observed in these mice.

In addition to previous findings that ERAP1^−/−^ mice have exaggerated innate and adaptive immune responses to a variety of stimuli^9,10,34–36^, H&E staining revealed evidence of severe lumbo-sacral IVD degeneration in ERAP1^−/−^ mice, with disruption of local structures and the presence of mononuclear cellular infiltrations in the NP. Immunohistochemical analysis of the ERAP1^−/−^ spines revealed that mononuclear infiltrates stained positively for TNFα and IL-23 in the cytoplasm. Both of these cytokines are known to play an important role in AS^42^. TNFα is thought to play a major role in disc degeneration via recruitment of inflammatory cells to the IVD, destruction of extracellular matrix, and calcification and hyperalgesia associated with IVD disease^43^. The IVD infiltrating cells also stained positive for IgM deposits and F4/80 + macrophages, both of which have been previously identified in vertebral biopsies derived from AS patients^39^. Proinflammatory cytokines and macrophage infiltration at the NP are likely responsible for the disc degeneration observed in ERAP1^−/−^ mice. It is possible that inflammation observed at the L6/S1 joint is in response to normal mechanical stress in the axial skeleton,^43^ with exaggerated immune responses caused by ERAP1-dependent Tr1 reduction^10^.

Commensal bacteria have been shown to play an important role in shaping the immune system, maintaining skeletal and gut homeostasis, and are thought to contribute to inflammatory diseases such as Rheumatoid Arthritis (RA), IBD, and MS^15–17,44^. Using 16S rRNA fecal phenotyping we determined that ERAP1^−/−^ mice spontaneously developed a significant fecal overabundance of specific bacterial species, including the *Cyanobacteria* and *Actinobacteria* phyla; as wells as *Prevotella*, *Odoribacter*, *Bacteroides*, *YS2, Clostridiales* and *Parabacteroides* genera. In addition, ERAP1^−/−^ mice had a significant deficiency of genera *Lachnospiraceae*, *Christensenellaceae* and *S24.7*. Overabundance of *Prevotella* has been previously reported in HLA-B27 transgenic rats^24^, which, similarly to our ERAP1^−/−^ mice develop osteoporosis^45^. *Prevotella* has also been reported to be enriched in the terminal ileum of AS patients^14^. Similarly to our results, increased *Bacteroides* has been reported to be increased in the fecal samples of AS patients^46^. While survey of the terminal ileum of AS patients revealed increases in *Lachnospiraceae*, *Ruminococcaceae* and reduction in *Streptococcus* and *Actinomyces* genera^14^, and Firmicutes phylum^47^, we did not observe such trends in the ERAP1^−/−^ mice. Since the data on human intestinal microbiome in AS patients is limited, future, larger studies are warranted to expand the knowledge of microbiome influences on human AS. ERAP1^−/−^ mice are a suitable model for investigating interactions between gut dysbiosis, skeletal and immune systems.

We and others have shown that ERAP1 alters the selection of immunodominant T cell epitopes and antigen specific T cell functions^34–37^. ERAP1’s role in immunodominance may also be responsible for the gut dysbiosis observed in ERAP1^−/−^ mice due to altered immune tolerance allowing for aberrant microbial communities to colonize the intestines of ERAP1^−/−^ mice. Cross-fostered ERAP1^−/−^ mice did not resolve spinal inflammation or reduce the severity of ankylosis and osteoporosis, downplaying the role of the intestinal microbiota in these phenotypes. We must note, however, that because *Bacteroides*, *Parabacteroides* and *Christensenellaceae* genera levels were not normalized in cross-fostered ERAP1^−/−^ mice, it is possible that these commensal microbes play a role in the skeletal phenotypes observed in ERAP1^−/−^ mice. While relationship between microbial communities (such as *Bacteroides fragilis*^48^ and *Clostridium* species^17^) and Treg development and function has been shown, our work also suggests a possible link between dysbiosis and Tr1 cells. Future studies dissecting relationship between dysbiosis and Tr1 cells are justified.

In summary, we showed that ERAP1^−/−^ mice closely mimic intestinal^4,14^, immune^39,42^ and skeletal^4^ features of AS and may serve as a useful model for studying pathogenesis of AS. We also determined that ERAP1 is important for Tr1 cell production. Interestingly, defects in Tr1 cell numbers and functions have been implicated in IDDM^27^, MS^28^, and IBD^29^. Given these associations, further studies investigating the role of ERAP1 in Tr1 cell differentiation and function may prove to be useful in understanding if ERAP1-mediated Tr1 reduction plays a role in pathogenesis of AS or any other ERAP1-associated diseases^30^.

## Materials and Methods

### Animal Procedures

All experiments were performed on C57BL/6 (WT) or C57BL/6 global ERAP1 knockout mice (ERAP1^−/−^), which were a kind gift from Dr. Kenneth Rock (Professor, University of Massachusetts Medical School). All mice used in experiments were bred in house. All animal procedures were reviewed and approved by the Michigan State University EHS, IBC, and IACUC (http://iacuc.msu.edu.proxy1.cl.msu.edu/) and conformed to NIH guidelines (AUF number: 02/13-045-00). Care for mice was provided in accordance with PHS and AAALAC standards.

### µCT Imaging and Ankylosis Scoring

Vertebra were harvested, fixed, and scanned using a GE Explore Locus microcomputed tomography (μCT) system as described previously^49^. Scans were performed using 20 μm voxel resolution obtained from 720 views. Beam angle increment of 0.5, beam strength of 80 peak kV and 450 µA were used. Each run included age-matched WT, and ERAP^−/−^ spines, and a calibration phantom for standardization and consistency. Isosurface images were created using an averaged autothreshold value of 1009, to separate bone from soft tissue using MicroView software. Right and left sides of the lumbar vertebrae 6 (L6) were scored separately and the scores were averaged to calculate the Mean Ankylosis Score. A “0” represented WT morphology of L6; a 1 represented the beginning of a syndesmophyte growing from L6 0.5–1 mm in length; a 2 represented a syndesmophyte extending 1–2 mm; a 3 represented a bridging syndesmophyte extending over 2 mm and contacting the iliac bone, but not fused with S1; and a 4 represented a fully fused joint. Representative scores shown in Fig. 1a.

Trabecular bone analysis of the S1 vertebra was performed using advanced ROI generated using splines in the transverse plane outlining the entire length of the vertebral body excluding the outer cortical bone. Average threshold values of 1232 for sacrum were used in order to separate bone from bone marrow. Bone mineral density, content, bone volume fraction, trabecular thickness, spacing and number values, as well as the representative isosurface images were generated using GE Healthcare MicroView software. Bone measurements were blinded until the final analysis.

### AS Index

AS index was calculated by taking the sum of the Mean Ankylosis Score and an assigned BMD score, where 325–300 mg/cc received score 1, 299–275–2, 274–250–3 and <250–4.

### Erosion Scoring

SI joints were assessed and scored for erosions using MicroView software in sagittal plane, where “0” represented a smooth joint; “1” represented a joint with mild erosions; and “2” represented erosions >1 mm in size or >3 erosions in one joint. Both SI joints were scored separately and averaged.

### DSS-Induced Colitis Experiments

8-week-old male mice were treated with 3% DSS (MP Biologicals) dissolved in sterile distilled water. Mice were given DSS in sterile drinking water ad libitum for the experimental days 1–7 (controls received sterile water only) followed by regular water until the end of the study. The DSS solutions were made fresh on day 0 and 3. Body weight, stool consistency, and the presence of blood in the stool were determined daily, as previously described^50^. The baseline clinical score was determined on day 0. Scoring procedures were performed, as follows. Weight loss relative to baseline: 0, no weight loss; 1, 1–5% weight loss; 2, 5–10% weight loss; 3, 10–20% weight loss; and 4, >20% weight loss. Stool scores were determined as follows: 0, well-formed pellets; 1, semiformed stools that did not adhere to the anus; 2, pasty and semiformed stools that adhered to the anus; 3, liquid stools that adhered to the anus. Bleeding scores were determined as follows: 0, no blood; 1, minor blood traces in stool visible; 2, visible blood traces in stool; 3, visible blood traces and dark stool with no rectal bleeding; 4, gross rectal bleeding. DAI was calculated by the addition of body weight, stool consistency, and rectal bleeding scores and dividing the total number by 3.

### Microbial Community Analysis

Fecal samples were shipped to Microbiome Insights company on dry ice, who performed the sequencing and analysis. 16S (V4 region) genes were sequenced on an Illumina MiSeq. Raw Fastq files were quality-filtered and clustered into 97% similar OTUs using the mothur software pipeline [http://www.mothur.org]. 3.23428 × 10^5^ high-quality bacterial reads were obtained. The final dataset had 3469 OTUs (including those occurring once with a count of 1) and a read range of 1.1499 × 10^4^ and 1.9824 × 10^4^. High quality reads were classified using Greengenes (v. 18_9) as the reference database. OTU abundances were aggregated into genera and plotted as the relative abundances. OTU abundances were summarized with the Bray-Curtis index and a non-metric multidimensional scaling (NMDS) analysis was performed to visualize microbiome similarities in ordination plots. Permutational multivariate analysis of variance (PERMANOVA) with Bonferroni correction was used to calculate significant differences between groups.

### Histology

Colon sections were harvested from DSS-treated WT or ERAP1^−/−^ mice at day 7, fixed, stained with hematoxylin and eosin (H&E) and scored, as previously described^22^. Mice received a score of 0–3 for inflammation with 1, 2, and 3 corresponding to lymphocytic infiltration of the epithelium, mucosa, and transmural inflammation, respectively. Colons were then scored 0–3 for pathologic changes to intestinal architecture with 1, 2, and 3 corresponding to focal erosions, extensive erosions, and extensive ulcerations with granulation tissue, respectively.

Spines were harvested, fixed for 48–72, hours and further processed for H&E and immunohistochemistry as described below.

### Routine Hematoxylin and Eosin Stain

Tissue samples previously fixed in 10% Neutral Buffered Formalin were processed and vacuum infiltrated with paraffin on the Sakura VIP 2000 tissue processor; followed by embedding with the ThermoFisher HistoCentre III embedding station. Once blocks were cooled, excess paraffin was removed from the edges, placed on a Reichert Jung 2030 rotary microtome and faced to expose tissue sample. Once the block is faced it is cooled and finely sectioned at 4–5 microns. Sections were dried in a 56 °C slide incubator to ensure adherence to the slides for 2–24 hours not exceeding this temperature which would potentially destroy antigenic components. Slides were removed from the incubator and stained with a routine Hematoxylin and Eosin method as follows: Two changes of Xylene–5 minutes each, two changes of absolute ethanol–2 minutes each, two changes of 95% ethanol–2 minutes each, running tap water rinse for 2 minutes, endure Hematoxylin (Cancer Diagnostics – Durham, NC) for 1 ½ minutes followed directly by a 10–15 second differentiation in 1% aqueous glacial acetic acid and running tap water for 2 minutes to enhance nuclear detail. Upon completion of running tap water slides were placed in one change of 95% ethanol–2 minutes, 1% Alcoholic Eosin-Phloxine B–2 minutes to stain cytoplasm, one change of 95% ethanol for 2 minutes, four changes of 100% ethanol–2 minutes each, four changes of Xylene–2 minutes each followed by coverslipping with synthetic mounting media for permanent retention and visualization with light microscopy.

### Immunohistochemistry – IL23, F4/80, IgM, TNFα Primary Antibodies

Specimens were decalcified in 14% EDTA, processed, embedded in paraffin and sectioned on a rotary microtome at 4μs. Sections were placed on slides coated with 2% 3-Aminopropyltriethoxysilane and dried at 56 °C overnight. The slides were subsequently deparaffinized in Xylene and hydrated through descending grades of ethyl alcohol to distilled water. Slides were placed in Tris Buffered Saline pH 7.4 (Scytek Labs – Logan, UT) for 5 minutes for pH adjustment. Following TBS, slides underwent heat induced epitope retrieval utilizing Scytek Citrate Plus Retrieval pH 6.0 (see Supplemental Table 2) or Enzyme Retrieval (see Supplemental Table 2) followed by rinses in several changes of distilled water. Endogenous Peroxidase was blocked utilizing 3% Hydrogen Peroxide/Methanol bath for 30 minutes followed by running tap and distilled water rinses. Following pretreatments standard micro-polymer complex staining steps were performed at room temperature on the IntelliPath™ Flex Autostainer. All staining steps were followed by rinses in TBS Autowash buffer (Biocare Medical – Concord, CA). After blocking for non-specific protein with Rodent Block M (Biocare) for 5 or 10 minutes; sections were incubated with specific primaries (see Supplemental Table 2) in normal antibody diluent (NAD-Scytek) and incubated 60 minutes. Micro-Polymer (Biocare) reagents were subsequently applied for specified incubations (see Supplemental Table 2) followed by reaction development with Romulin AEC™ (Biocare) (see Supplemental Table 2) and counterstained with Cat Hematoxylin (see Supplemental Table 2).

### Isolation of Lymphocytes from Spleen and Lymph nodes

Spleen or Lymph node tissues were physically disrupted (by passage through a 40 µm sieve), followed by RBCs lysis by using 2 ml of ACK lysis buffer (Invitrogen) per sample. Cells were subsequently washed twice with complete RPMI medium (RPMI 1640 (Invitrogen) supplemented with 10% FBS, 1% PSF (penicillin, streptomycin, fungizone)), resuspended, and counted.

### Flow cytometry

For surface staining, 2 million cells were incubated with Fcγ block (BD Biosciences) and with the appropriate antibodies on ice for 45 minutes, and washed twice with FACS buffer. For intranuclear staining, following surface staining and washing, 4 million cells were fixed and stained with intranuclear antibodies using BD Pharmingen Transcription Factor Buffer Set (BD Bioscience) per manufacturer’s protocol.

For intracellular staining, 4 million splenocytes were plated in a 96-well plate in presence of 50 ηg/mL PMA (Calbiochem) and 1 µg/mL Ionomycin (Calbiochem) for 30 min, after which 1 µL/well of GolgiPlug (BD Biosciences) was added, and cells were incubated for additional 5.5 hours. Cells were washed twice with FACS buffer and surface stained as above, after which BD Cytofix/Cytoperm Fixation/Permeabilization kit (BD Biosciences) was used per manufacturer’s protocol.

List of antibodies used: PECy7-anti-CD4 (BD Biosciences), APC-Eflur780-anti-CD3 (eBioscience), Alexa488-anti-CD25 (Invitrogen), Pacific Blue-anti-Foxp3 (Invitrogen), APC-anti-IL-10 (BioLegend), PECy7-anti-CD49b (Invitrogen), PE-anti-Lag3 (BioLegend), PE-anti-F4/80 (eBioscience), Pacific Blue-anti-CD8 (BD Biosciences), APC-Cy7-anti-CD11b (BD Biosciences), PECy7-anti-CD11c (BD Biosciences), BV650-anti-Qa2 (BD Biosciences), FITC-anti-CD45RB (BD Biosciences), all at 4 μg/ml. Live/dead Fixable Aqua Dead Cell Stain Kit (Invitrogen) was used per manufacturer’s protocol to exclude dead cells from analysis.

Data were collected on BD LSR II instrument and analyzed using FlowJo software (Tree Star).

### Tr1 differentiation assay

Splenocytes and lymphocytes were isolated from the spleens and lymph nodes of 4 WT and 4 ERAP1^−/−^ mice. Cells from both tissues were processed into a homogenous single-cell suspension and combined. Naïve CD4^+^ T cells were isolated from this cell mixture using the MACS Mouse Naïve CD4^+^ T cell Isolation Kit (Miltenyi Biotec) per manufacturer’s instructions. Cells were plated in complete RPMI medium at 1 × 10^5^ cells/well in a 96-well high binding plate pre-coated with 2 µg/mL anti-CD3 mAb (BioLegend) and 2 µg/mL anti-CD28 mAb (BioLegend). IL-27 (Peprotech) was added to indicated wells at 25 ng/mL. Supernatant was collected from cultures at 96 hours at which point the media was replaced with media containing 0.1 µg/mL PMA (Calbiochem), 1 µg/mL Ionomycin (Calbiochem), 0.2 µL/well of GolgiPlug (BD Biosciences) and 1.95 µM Monesin (Sigma Aldrich). After 5 hours the cells were collected, washed and stained.

### Cross-fostering experiments

ERAP1^−/−^ and WT breeding cages were set up simultaneously to synchronize pregnancies. Upon showing physical signs of pregnancy, dams were removed from sires and housed separately. Within 48 hours after birth, ERAP1^−/−^ pups were transferred to lactating WT dams (CF ERAP1^−/−^), and vice versa, WT pups were transferred to ERAP1^−/−^ dams (CF WT). Control cages where WT pups were transferred to different WT dams and ERAP1^−/−^ pups were transferred to different ERAP1^−/−^ dams were also set up. Pups were weaned by 28 days of age. At 14 weeks of age mice were sacrificed and their spines were harvested and fixed for µCT and histology analysis. Average threshold value of WT control mice of 948 was used for trabecular bone analysis of S1 of cross-fostered and control animals using µCT. Fecal samples were collected at 4 and 14 weeks of age and frozen in liquid nitrogen.

### Statistics

All statistical analysis was performed using GraphPad Prism 7 software. Statistical tests performed for each data set and significance levels are indicated in the figure legends.

## Electronic supplementary material

Supplemental figures

## References

[1] Dean LE (2014). Global prevalence of ankylosing spondylitis. Rheumatology (Oxford).

[2] Braun J, Sieper J (2007). Ankylosing spondylitis. Lancet.

[3] Klingberg E (2012). Osteoporosis in ankylosing spondylitis - prevalence, risk factors and methods of assessment. Arthritis Res Ther.

[4] El Maghraoui A (2011). Extra-articular manifestations of ankylosing spondylitis: prevalence, characteristics and therapeutic implications. Eur J Intern Med.

[5] Brewerton DA (1973). Ankylosing spondylitis and HL-A 27. Lancet.

[6] Reveille JD (2012). Genetics of spondyloarthritis–beyond the MHC. Nat Rev Rheumatol.

[7] Cortes A (2015). Major histocompatibility complex associations of ankylosing spondylitis are complex and involve further epistasis with ERAP1. Nat Commun.

[8] Evans DM (2011). Interaction between ERAP1 and HLA-B27 in ankylosing spondylitis implicates peptide handling in the mechanism for HLA-B27 in disease susceptibility. Nat Genet.

[9] Aldhamen YA (2015). Autoimmune disease-associated variants of extracellular endoplasmic reticulum aminopeptidase 1 induce altered innate immune responses by human immune cells. J Innate Immun.

[10] Aldhamen YA (2013). Endoplasmic reticulum aminopeptidase-1 functions regulate key aspects of the innate immune response. PLoS One.

[11] Goto Y, Ogawa K, Hattori A, Tsujimoto M (2011). Secretion of endoplasmic reticulum aminopeptidase 1 is involved in the activation of macrophages induced by lipopolysaccharide and interferon-gamma. J Biol Chem.

[12] Ciccia F, Rizzo A, Triolo G (2016). Subclinical gut inflammation in ankylosing spondylitis. Curr Opin Rheumatol.

[13] Thjodleifsson B, Geirsson AJ, Bjornsson S, Bjarnason I (2007). A common genetic background for inflammatory bowel disease and ankylosing spondylitis: a genealogic study in Iceland. Arthritis Rheum.

[14] Costello ME (2015). Brief Report: Intestinal Dysbiosis in Ankylosing Spondylitis. Arthritis Rheumatol.

[15] Jethwa H, Abraham S (2017). The evidence for microbiome manipulation in inflammatory arthritis. Rheumatology (Oxford).

[16] Yadav SK (2017). Gut dysbiosis breaks immunological tolerance toward the central nervous system during young adulthood. Proc Natl Acad Sci USA.

[17] Atarashi K (2011). Induction of colonic regulatory T cells by indigenous Clostridium species. Science.

[18] Roncarolo MG, Gregori S, Bacchetta R, Battaglia M (2014). Tr1 cells and the counter-regulation of immunity: natural mechanisms and therapeutic applications. Curr Top Microbiol Immunol.

[19] Wakkach A (2003). Characterization of dendritic cells that induce tolerance and T regulatory 1 cell differentiation *in vivo*. Immunity.

[20] Araki A (2005). MyD88-deficient mice develop severe intestinal inflammation in dextran sodium sulfate colitis. J Gastroenterol.

[21] Zaki MH, Vogel P, Body-Malapel M, Lamkanfi M, Kanneganti TD (2010). IL-18 production downstream of the Nlrp3 inflammasome confers protection against colorectal tumor formation. J Immunol.

[22] Erben U (2014). A guide to histomorphological evaluation of intestinal inflammation in mouse models. Int J Clin Exp Pathol.

[23] Ni J, Wu GD, Albenberg L, Tomov VT (2017). Gut microbiota and IBD: causation or correlation?. Nat Rev Gastroenterol Hepatol.

[24] Lin P (2014). HLA-B27 and human beta2-microglobulin affect the gut microbiota of transgenic rats. PLoS One.

[25] Daft JG, Ptacek T, Kumar R, Morrow C, Lorenz RG (2015). Cross-fostering immediately after birth induces a permanent microbiota shift that is shaped by the nursing mother. Microbiome.

[26] Glatigny S (2012). Proinflammatory Th17 cells are expanded and induced by dendritic cells in spondylarthritis-prone HLA-B27-transgenic rats. Arthritis Rheum.

[27] Yu H (2017). Intestinal type 1 regulatory T cells migrate to periphery to suppress diabetogenic T cells and prevent diabetes development. Proc Natl Acad Sci USA.

[28] Astier AL, Hafler DA (2007). Abnormal Tr1 differentiation in multiple sclerosis. J Neuroimmunol.

[29] Groux H (1997). A CD4+T-cell subset inhibits antigen-specific T-cell responses and prevents colitis. Nature.

[30] Fierabracci A, Milillo A, Locatelli F, Fruci D (2012). The putative role of endoplasmic reticulum aminopeptidases in autoimmunity: insights from genomic-wide association studies. Autoimmun Rev.

[31] Chihara N, Madi A, Karwacz K, Awasthi A, Kuchroo VK (2016). Differentiation and Characterization of Tr1 Cells. Curr Protoc Immunol.

[32] Gregori S, Magnani CF, Roncarolo MG (2009). Role of human leukocyte antigen-G in the induction of adaptive type 1 regulatory T cells. Hum Immunol.

[33] Gregori S (2010). Differentiation of type 1 T regulatory cells (Tr1) by tolerogenic DC-10 requires the IL-10-dependent ILT4/HLA-G pathway. Blood.

[34] Rastall DP, Aldhamen YA, Seregin SS, Godbehere S, Amalfitano A (2014). ERAP1 functions override the intrinsic selection of specific antigens as immunodominant peptides, thereby altering the potency of antigen-specific cytolytic and effector memory T-cell responses. Int Immunol.

[35] Seregin SS (2013). Endoplasmic reticulum aminopeptidase-1 alleles associated with increased risk of ankylosing spondylitis reduce HLA-B27 mediated presentation of multiple antigens. Autoimmunity.

[36] York IA, Brehm MA, Zendzian S, Towne CF, Rock KL (2006). Endoplasmic reticulum aminopeptidase 1 (ERAP1) trims MHC class I-presented peptides *in vivo* and plays an important role in immunodominance. Proc Natl Acad Sci USA.

[37] Rastall DPW (2017). Mice expressing human ERAP1 variants associated with ankylosing spondylitis have altered T-cell repertoires and NK cell functions, as well as increased in utero and perinatal mortality. Int Immunol.

[38] Shido F (2006). Endoplasmic reticulum aminopeptidase-1 mediates leukemia inhibitory factor-induced cell surface human leukocyte antigen-G expression in JEG-3 choriocarcinoma cells. Endocrinology.

[39] Bron JL, de Vries MK, Snieders MN, van der Horst-Bruinsma IE, van Royen BJ (2009). Discovertebral (Andersson) lesions of the spine in ankylosing spondylitis revisited. Clin Rheumatol.

[40] Ciccia F (2010). Expansion of intestinal CD4+CD25(high) Treg cells in patients with ankylosing spondylitis: a putative role for interleukin-10 in preventing intestinal Th17 response. Arthritis Rheum.

[41] Guo H (2016). Functional defects in CD4(+) CD25(high) FoxP3(+) regulatory cells in ankylosing spondylitis. Sci Rep.

[42] Zambrano-Zaragoza JF, Agraz-Cibrian JM, Gonzalez-Reyes C, Duran-Avelar Mde J, Vibanco-Perez N (2013). Ankylosing spondylitis: from cells to genes. Int J Inflam.

[43] Molinos M (2015). Inflammation in intervertebral disc degeneration and regeneration. J R Soc Interface.

[44] Yan J, Charles JF (2017). Gut Microbiome and Bone: to Build, Destroy, or Both?. Curr Osteoporos Rep.

[45] Rauner M (2015). Loss of bone strength in HLA-B27 transgenic rats is characterized by a high bone turnover and is mainly osteoclast-driven. Bone.

[46] Stebbings S (2002). Comparison of the faecal microflora of patients with ankylosing spondylitis and controls using molecular methods of analysis. Rheumatology (Oxford).

[47] Gill T, Asquith M, Rosenbaum JT, Colbert RA (2015). The intestinal microbiome in spondyloarthritis. Curr Opin Rheumatol.

[48] Round JL, Mazmanian SK (2010). Inducible Foxp3+regulatory T-cell development by a commensal bacterium of the intestinal microbiota. Proc Natl Acad Sci USA.

[49] Irwin R, Lee T, Young VB, Parameswaran N, McCabe LR (2013). Colitis-induced bone loss is gender dependent and associated with increased inflammation. Inflamm Bowel Dis.

[50] Lee T (2013). beta-Arrestin-1 deficiency protects mice from experimental colitis. Am J Pathol.

