# Supplementary material for: ERAP1 deficient mice have reduced Type 1 regulatory T cells and develop skeletal and intestinal features of Ankylosing Spondylitis
Source: Sci Rep. 2018 Aug 20;8:12464. doi: 10.1038/s41598-018-30159-5 (PMC6102283; doi:10.1038/s41598-018-30159-5)
Supplement: Supplementary file 1 — Supplemental figures [file 41598_2018_30159_MOESM1_ESM.pdf]

ERAP1 deficient mice have reduced Type 1 regulatory T cells and develop skeletal and intestinal features of Ankylosing Spondylitis.

Yuliya Pepelyayeva,[1†] David P.W. Rastall (D.O., Ph.D.),[1†] Yasser A. Aldhamen (Ph.D.),[1] Patrick O'Connell,[1] Sandra Raehtz (D.O., Ph.D.),[3] Fadel S. Alyaqoub (Ph.D.),[1] Maja K. Blake,[1] Ashley M. Raedy,[1] Ariana M. Angarita,[1] Abdulraouf M. Abbas,[1] Cristiane N. Pereira-Hicks,[1] Sarah G. Roosa,[1] Laura McCabe (Ph.D.),[3,4,5] Andrea Amalfitano (D.O., Ph.D.),[1,2]*

[1]Department of Microbiology and Molecular Genetics, [2]Department of Pediatrics, College of Osteopathic Medicine, [3]Department of Physiology; [4]Department of Radiology, and [5]Biomedical Imaging Research Center, Michigan State University, East Lansing, MI 48824

† Denotes equal contribution of coauthors

* Denotes corresponding author

Supplemental Figures

Supplemental Figure 1

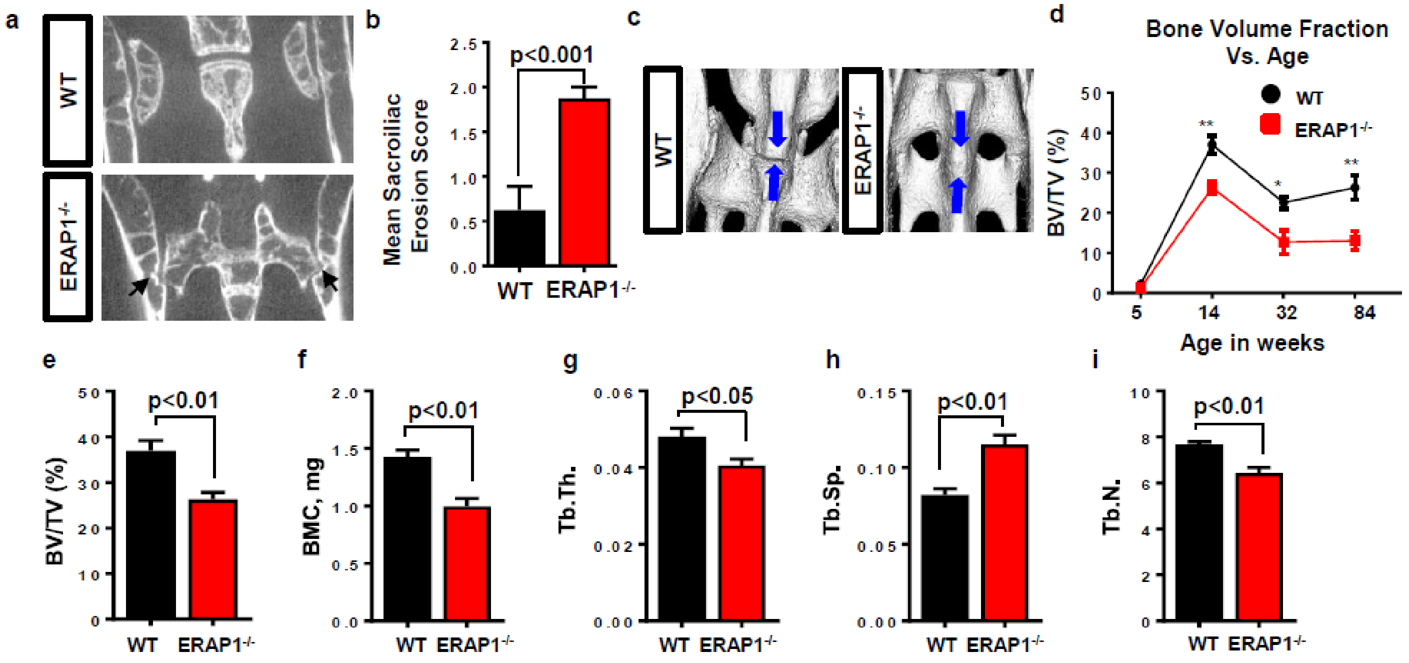

Supplemental Figure 1. µCT analysis of sacral region. The spines from 84-week-old female ERAP1$^{-/-}$ and WT female mice (n=8) were harvested and analyzed with µCT. **(a)** Representative 2D radiograph demonstrating erosions **(black arrows)** at the SI joint. **(b)** SI joints were assessed for erosions bilaterally, averaged per side and graphed. **(c)** Representative images demonstrating calcification of the anterior longitudinal ligament between L6 and S1 in ERAP1$^{-/-}$ animals, depicted with **blue arrows**. The spines from 14-week-old ERAP1$^{-/-}$ and WT female mice (n=5) were harvested and analyzed with µCT. **(d)** Graph comparing BV/TV% of the trabecular bone of S1 vertebra between WT and ERAP1$^{-/-}$ mice at various ages. Bar graphs demonstrating BV/TV **(e)**, BMC **(f)**, Tb.Th. **(g)**, Tb.Sp. **(h)**, and Tb.N. **(i)** of the trabecular bone of S1 vertebra. Values on graphs are the mean ± SEM. p values calculated by student's t-test, where * - $p<0.05$ and ** - $p<0.01$ compared to age-matched WT mice.

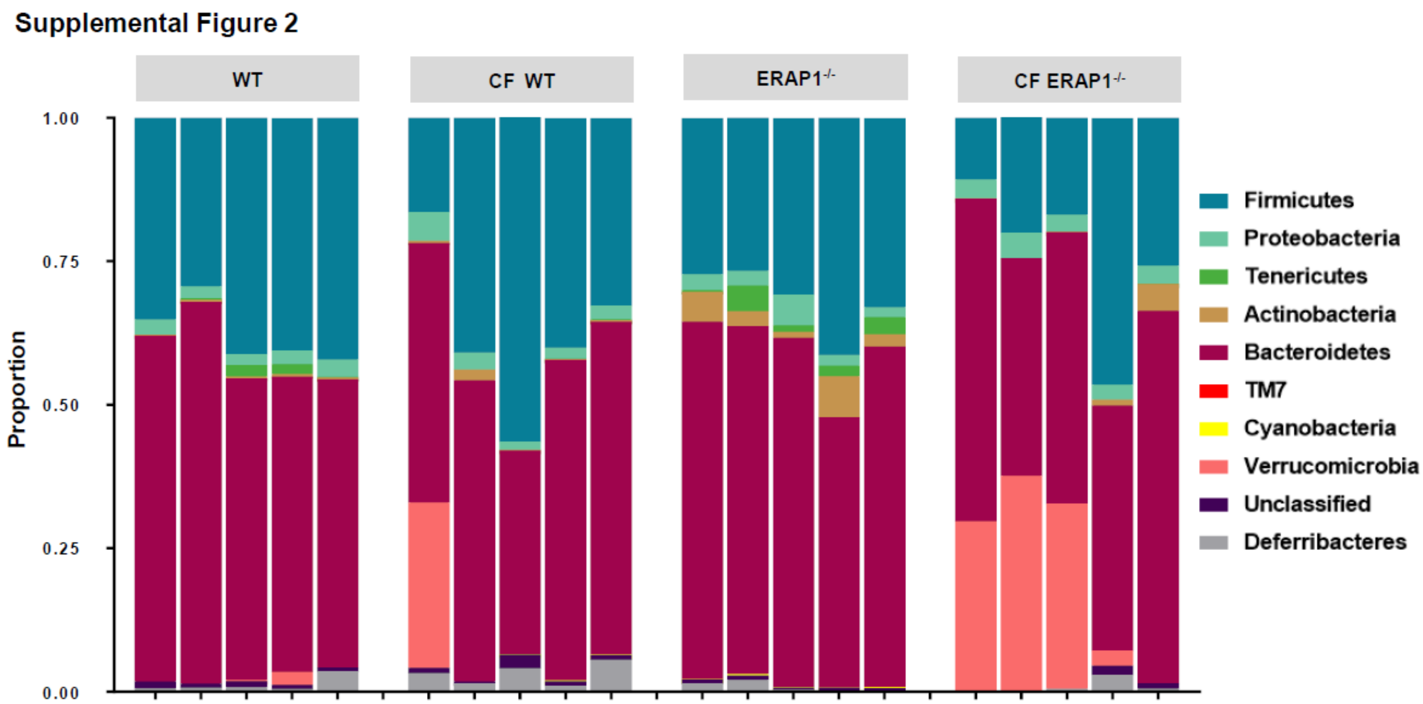

Supplemental Figure 2. Relative abundance of phyla in WT, CF WT, ERAP1$^{-/-}$ and CF ERAP1$^{-/-}$ fecal samples. 16s rRNA phenotyping was performed with illumina on fecal samples of 14-week-old male and female mice (n=5). Stacked bar graph showing relative abundance of phyla in fecal samples of WT, CF WT, ERAP1$^{-/-}$, and CF ERAP1$^{-/-}$ mice.

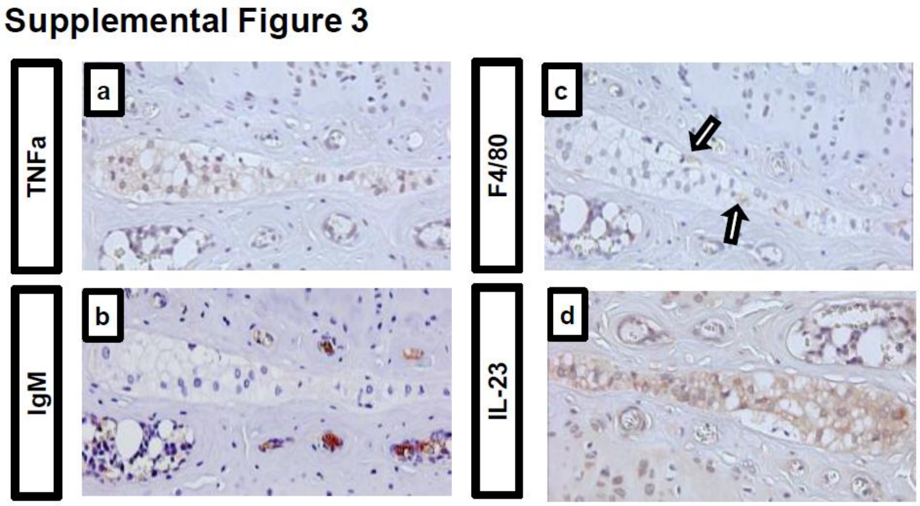

Supplemental Figure 3. Histopathological analysis of the ankylosed intervertebral joints from CF ERAP1$^{-/-}$ mice. 14-week-old cross-fostered ERAP1$^{-/-}$ female and male mice were

sacrificed. Their spines were harvested, fixed, decalcified and stained. Representative immunohistochemistry images of L6/S1 intervertebral disc at 20X magnification for TNFα **(a)**, IgM **(b)**, F4/80 **(c)**, with **arrows** pointing at F4/80+ macrophages, and IL-23 **(d)**.

Supplemental Table 1

Supplemental Table 1. Genera that were significantly different between WT and ERAP1$^{-/-}$ mice.

Increased in ERAP1$^{-/-}$			
Genus	WT vs ERAP1$^{-/-}$	ERAP1$^{-/-}$ vs CF ERAP1$^{-/-}$	WT vs CF ERAP1$^{-/-}$
Bacteroides	****	NS	**
Clostridiales_unclassified	*	**	NS
Odoribacter	***	***	NS
Parabacteroides	*	NS	*
Prevotella	****	*	NS
YS2_unclassified	*	*	NS

Decreased in ERAP1$^{-/-}$			
Genus	WT vs ERAP1$^{-/-}$	ERAP1$^{-/-}$ vs CF ERAP1$^{-/-}$	WT vs CF ERAP1$^{-/-}$
Christensenellaceae_unclassified	*	NS	*
Lachnospiraceae_unclassified	**	NS	NS
S24.7_unclassified	*	NS	NS

Table summarizing differences of the genus-level OTUs that were found to be significantly increased (top half of the table) or decreased (bottom half of the table) in fecal samples of ERAP1$^{-/-}$ mice compared to WT. Differences between ERAP1$^{-/-}$ and CF ERAP1$^{-/-}$ and WT and CF ERAP1$^{-/-}$ mice are summarized in columns 2 and 3, respectively, with arrows representing direction of change in CF ERAP1$^{-/-}$ fecal samples. p values calculated by student's t-test, where * - p<0.05, ** - p<0.01, *** - p<0.001, **** - p<0.0001 and NS - not significant.

Supplemental Table 2. Details of antibodies used for Immunohistochemistry staining.

Primary Antibody	Ab Vendor:	Pretreatment:	Primary:	Staining System:
Rabbit anti – IL23 Polyclonal	Abcam #115759 Cambridge, MA	0.03% Pronase E in TBS for 10 minutes at 37°C	1:100 in NAD – 2 Hours	ProMark Rabbit on Rodent HRP Polymer™ - 30 minutes<br>AEC – 5 minutes<br>CATHE Hematoxylin 1:10 – 1 minute
Rat anti – F4/80 Monoclonal	Bio-Rad # *MCA497G* Hercules, CA	Overnight Retrieval overnight at 56°C	1:100 in NAD Overnight	ProMark Rat on Mouse HRP Polymer™<br>15 minutes – Probe<br>15 minutes – Polymer<br>AEC – 5 minutes<br>CATHE Hematoxylin 1:10 – 1 minute
Rabbit anti – IgM Polyclonal	ThermoFisher *#61-6800 Rockford, IL*	Overnight Retrieval overnight at 56°C	1:300 in NAD – 60 minutes	ProMark Rabbit on Rodent HRP Polymer™ - 30 minutes<br>AEC – 5 minutes<br>CATHE Hematoxylin 1:5 – 5 minutes
Rabbit anti-TNF alpha Polyclonal	Abcam #6671 Cambridge, MA	Overnight Retrieval overnight at 56°C	1:100 in NAD – 2 Hours	ProMark Rabbit on Rodent HRP Polymer™ - 30 minutes<br>AEC – 5 minutes<br>CATHE Hematoxylin 1:10 – 1 minute
